# Arginine Promoted Ovarian Development in Pacific White Shrimp *Litopenaeus vannamei* via the NO-sGC-cGMP and TORC1 Signaling Pathways

**DOI:** 10.3390/ani14131986

**Published:** 2024-07-05

**Authors:** Xin Zhang, Yanan Yin, Haitao Fan, Qicun Zhou, Lefei Jiao

**Affiliations:** School of Marine Sciences, Ningbo University, Ningbo 315211, China18272573868@163.com (Y.Y.);

**Keywords:** ovarian development, vitellogenin synthesis, crustacean feeds, arginine, *Litopenaeus vannamei*

## Abstract

**Simple Summary:**

This study aimed to evaluate the effects of arginine on the ovarian development of Pacific white shrimp (*Litopenaeus vannamei*). The analyzed arginine supplementation levels in each diet were 2.90%, 3.58%, 4.08%, 4.53%, 5.04%, and 5.55%, respectively. The results showed that dietary arginine supplementation could enhance endogenous and exogenous vitellogenin syntheses to promote ovary development in *L. vannamei*, and the appropriate dosages were 4.08% and 4.53%. The NO-sGC-cGMP and mTORC1 signaling pathways mediated arginine in the regulation of ovary development in *L. vannamei*. This finding could offer valuable theoretical knowledge and be a helpful reference for future investigations into shrimp broodstock nutrition and artificial broodstock diets.

**Abstract:**

This study aimed to evaluate the effects of arginine (0.5%, 1%, 1.5%, 2%, and 2.5% arginine supplementation levels were selected) on the ovarian development of Pacific white shrimp (*Litopenaeus vannamei*). The analyzed arginine supplementation levels in each diet were 2.90%, 3.58%, 4.08%, 4.53%, 5.04%, and 5.55%, respectively. A total of 540 shrimp (an initial weight of approximately 14 g) with good vitality were randomly distributed into six treatments, each of which had three tanks (300 L in volume filled with 200 L of water), with 30 shrimp per duplicate. Shrimp were fed three times a day (6:00 a.m., 11:00 a.m., and 6:00 p.m.). The results showed that after the 12-week raring cycle, shrimp fed with 4.08% and 4.53% Arg achieved better ovary development, which was identified by ovarian stage statistics, ovarian morphology observation, serum hormone levels (methylfarneside (MF); 5-hydroxytryptamine (5-HT); estradiol (E2); and gonadotropin-releasing hormone (GnRH)), gene expression (DNA meiotic recombinase 1 (*dmc1*), proliferating cell nuclear antigen (*pcna*), drosophila steroid hormone 1 (*cyp18a*), retinoid X receptor (*rxra*), and ecdysone receptor (*ecr*)). Further in-depth analysis showed that 4.08% and 4.53% Arg supplementation increased the concentration of vitellogenin in hepatopancreas and serum (*p* < 0.05) and upregulated the expression level of hepatopancreatic *vg* and *vgr* (*p* < 0.05), which promoted the synthesis of hepatopancreas exogenous vitellogenin and then transported it into the ovary through the vitellogenin receptor and further promoted ovarian maturation in *L. vannamei.* Meanwhile, compared with the control group, the expression level of *vg* in the ovary of the 4.53% Arg group was significantly upregulated (*p* < 0.05), which indicated endogenous vitellogenin synthesis in ovarian maturation in *L. vannamei.* Moreover, the expression of genes related to the mechanistic target of the rapamycin complex 1 (mTORC1) pathway and protein levels was regulated by dietary arginine supplementation levels. Arginine metabolism-related products, including nitric oxide synthase (NOS), nitric oxide (NO), and cyclic guanosine monophosphate (cGMP), were also affected. RNA interference was applied here to study the molecular regulation mechanism of arginine on ovarian development in *L. vannamei*. A green fluorescent protein (GFP)-derived double-stranded RNA (dsGFP) is currently commonly used as a control, while TOR-derived dsRNA (dsTOR) and NOS-derived dsRNA (dsNOS) were designed to build the TOR and NOS in vivo knockdown model. The results showed that the mTORC1 and NO-sGC-cGMP pathways were inhibited, while the vitellogenin receptor and vitellogenin gene expression levels were downregulated significantly in the hepatopancreas and ovary. Overall, dietary arginine supplementation could enhance endogenous and exogenous vitellogenin synthesis to promote ovary development in *L. vannamei*, and the appropriate dosages were 4.08% and 4.53%. The NO-sGC-cGMP and mTORC1 signaling pathways mediated arginine in the regulation of ovary development in *L. vannamei*.

## 1. Introduction

The Pacific white shrimp, *Litopenaeus vannamei* (Boone, 1931), is the top aquatic species of commercial importance worldwide, constituting more than 80% of global shrimp production. The global annual production of farmed shrimp recently reached more than 7.7 million tons, representing a value of more than USD 33 billion [1]. Reproduction is a critical step in shrimp culture, and good-quality parents are necessary to produce healthy shrimp larvae. Despite increasing research on the nutritional physiology and compound feed of shrimp, the cultivation of *L. vannamei* broodstock still relies on a combination of the artificial removal of the unilateral eye stalk and feeding a variety of fresh and frozen foods, such as silkworm, marine polychaete, squid, oysters, and mussels [2,3]. Given the risk of disease transmission and unstable nutritional quality, fresh food is not considered suitable for the sustainable development of shrimp broodstock farming. Therefore, it is necessary to conduct a nutritional study and develop a well-balanced and biosafe diet for *L. vannamei* broodstock [3]. However, there are few studies on the nutritional regulation of ovarian development in *L. vannamei* broodstock.

Nutrition is essential for sustaining the growth and reproduction of any organism, as nutrients not only provide the basis for the growth of cells, organs, and the entire body but also regulate the development of reproductive organs and the growth of the next generation. Among nutrients, amino acids are essential for the fertility of humans and all animals, which play vital roles in reproduction, development, and production [4]. In recent years, the regulatory role of arginine (Arg) in reproductive physiological functions has been gradually established with limited research. In livestock and poultry, Arg supplementation could alleviate the effects of dietary restriction on follicle development by enhancing glucose metabolism and cell proliferation in granulose cells and alleviating the abnormal estradiol secretion in the ≥2.5 mm follicles, accompanied by recovering the expressions of the neuropeptide VF precursor and gonadotropin-releasing hormone in the hypothalamus [5]. Supplementing 0.20% Arg levels in low-crude-protein diets influenced the ovary morphology of laying hens and regulated the expression of reproductive hormone genes in the hypothalamic–pituitary–gonadal axis [6]. In aquatic animals, two reports explained Arg’s promoting effect on ovary development in Chinese mitten crab and Nile tilapia [7,8]. The knowledge of Arg nutritional requirements in shrimp has to be obtained regarding shrimp species [9,10]. However, there is a lack of comprehensive information available regarding the specific effects of Arg on ovarian development in shrimp.

This study aimed to evaluate Arg’s influence on ovarian development in *L. vannamei* to gain extended insight into the current knowledge of Arg effects in shrimp species. The results of our study could potentially offer valuable theoretical knowledge and serve as a useful reference for future investigations into shrimp broodstock nutrition and artificial broodstock diets.

## 2. Materials and Methods

### 2.1. Experiment 1: 12-Week Arginine Feeding Experiment

#### 2.1.1. Farming Experiment

Six different diets, which contained varying Arg levels, were prepared for the experiment. The first group was fed the standard diet without any additional Arg (which served as the control group). The remaining five groups were given diets supplemented with 0.5%, 1%, 1.5%, 2%, and 2.5% Arg, respectively. The Arg was purchased from Shanghai Sanjie Bio-Tech Co., Ltd., Shanghai, China. The Arg composition of the diets in each treatment was measured using an L-8900 amino acid analyzer (Hitachi, Tokyo, Japan). The standard diet contained fish meal, krill meal, and other protein feed, which contained a certain proportion of Arg. Therefore, we measured the Arg concentration in each diet and found that the analyzed Arg levels were 2.90%, 3.58%, 4.08%, 4.53%, 5.04%, and 5.55%, respectively. Table 1 contains details on the constituents and ingredients of the basal diet. The crude protein, crude lipid, moisture, and ash content in diets were determined according to the Association of Official Analytical Chemists (2006) method. The feed ingredients acquired from Tianbang Feed Co., Ltd. (Ningbo, China) were crushed and screened through a 60-mesh sieve. The raw materials were measured based on the feed formula and mixed carefully using a mixing mixer. Afterward, the blended feed ingredients underwent extrusion processing, which involved passing through a twin-screw extruder (F-26, South China University of Technology, China) to produce extruded feed strips. The feed strips were transferred to a granulator (G-250, South China University of Technology, China) to produce particles with 1.5/2.5 mm diameters and lengths of 0.3–0.6 cm. These pellets were baked in a 60°C oven for drying. After that, the shrimp feed was vacuum-sealed and stored at −20 °C.

These pellets were baked in an oven at 60 °C for 2 h before being naturally dried. After that, the shrimp feed was vacuum-sealed and stored at −20 °C.

#### 2.1.2. Shrimp Culture and Condition Monitoring

Our experiment was conducted at Ningbo Marine Fishery Science and Technology Innovation Base. Before the experiment started, juvenile shrimp bought from Ningbo Yinzhou Jinghong Aquatic Products Professional Cooperative were cultured in a cement pond where the water flowed and aerated at the surrounding temperature. After 12 weeks of feeding, 540 shrimp (approximately 14 g initial weight) with good vitality were randomly divided into 18 tanks (300 L cylindrical fiber-glass tanks filled with 200 L of water), and each tank was set up of 30 shrimp. We counted 15 shrimp for each tank and weighed them and then selected the remaining 15 shrimp to make the final total weight around 420 g. The shrimp were fed 3 times a day (6:00 a.m., 11:00 a.m., and 6:00 p.m.), and the feed supply was 6–8% body weight/days early in the first 2 weeks and 3–5% body weight/days towards the end according to observations of feeding and feed waste. The shrimp were reared in water at 25.4–26.8 °C, 24.3–25.3 salinity, 7.6–7.8 pH, 5.0–5.6 mg/L dissolved oxygen, and 0.02–0.04 mg/L ammonia nitrogen, with 50% of the water in each tank being replaced each day before the first meal. Dead shrimp were removed and weighed once found.

#### 2.1.3. Sample Collection

After the 12-week experiment, each shrimp’s ovarian development stage was recorded. The method of ovarian staging identification we used has been referred to in previous reports [11,12]. Hemolymph samples were collected from 5 shrimp (at random) in each tank from the pericardial cavity. These samples were placed into 1.5 mL centrifuge tubes and left to cool at 4 °C overnight before being centrifuged (4000 rpm, 10 min). The hepatopancreas samples from 5 shrimp in each tank were mixed with 0.9% normal saline to create a homogeneous mixture. Then, they were centrifugated at 4000 rpm for 10 min at a temperature of 4 °C. The supernatant of hemolymph and hepatopancreas samples was pooled, transferred into a 200 μL Eppendorf tube, and stored at −80 °C for later hormone concentrations and enzyme activity, respectively. Ovary and hepatopancreas of 8 shrimp from each tank were collected and pooled for gene expression analysis, crude protein, and amino acids analysis. Ovaries from 2 shrimp in each tank were cut into small pieces and put into 4% paraformaldehyde for light microscopy observation.

### 2.2. Experiment 2: RNA Interference Experiment for Silencing TOR and NOS Genes

#### 2.2.1. Double-Stranded RNA (dsRNA) Preparation

Table 2 shows the design of specific primers for amplifying sense and anti-sense strands individually using polymerase chain reaction (PCR). These primers have a T7 RNA polymerase binding site at their 5′ terminus. The PCR product was assayed for concentration using 1.5% agarose gel electrophoresis, and the PCR product was purified from the gel (E.Z.N.A.Gel Extraction Kit-Spin Protocol). After purifying and quantifying the amplified products, we proceeded to transcribe single-stranded RNAs using T7 RNase polymerase (T7 RiboMAZ™ Express Large Scale RNA Production System, Promega, Madison, WI, USA) through an in vitro process. These single-stranded RNAs were combined and annealed to form double-stranded RNA (dsRNAs) according to the manufacturer’s instructions. Subsequently, gel electrophoresis and sequencing analysis confirmed the successful synthesis of dsRNAs (Supplementary Tables S1 and S2). The dsRNAs were purified, verified, quantified, and stored at −80 °C.

#### 2.2.2. RNA Interference Treatment

A total of 45 white shrimp (mean weight: 30 ± 0.41 g) were fed diets containing (54% protein, 9.5% lipid, and 4.53% Arg. Subsequently, shrimp with good viability and stage Ⅱ ovarian development were randomly divided into three groups and injected with dsRNA to ensure that the targeted genes were downregulated. During the entire trial, dsRNA solutions targeting specific gene signaling pathways (key target genes of signaling pathways) were prepared and injected into the ventral sinus at a concentration of 1 μg/g shrimp body weight. Shrimp in the control group were injected with a dsGFP solution.

Three groups were injected with dsTOR, dsGFP, and dsNOS for gene knockdown treatments. Following 48 h of dsRNA injection, RNA extraction of the hepatopancreas and ovary was performed on all shrimp. Three shrimp were mixed as one sample for analysis. Therefore, each group’s sample number was 5 (n = 5). The primers were designed to analyze the gene expression of *nos* and *tor* along with a reference gene to confirm the RNA interference efficiency.

### 2.3. Experimental Parameters

#### 2.3.1. Hormone Concentrations and Enzyme Activity

According to the manufacturer’s instructions, nitric oxide (NO) and NOS activities were determined using commercial kits (Nanjing Jiancheng Bioengineering Institute, Nanjing, China). 5-Hydroxytryptamine (5-HT), gonadotropin-releasing hormone (GnRH), estradiol (E2), vitellogenin (VTG), methylfarnesides (MF), and cyclic guanosine monophosphate (cGMP) concentrations were determined using commercial kits (Jiangsu Microplate Reagent Co., Ltd., Jiangsu, China) following the instructions provided by the manufacturer.

#### 2.3.2. Ovarian Histological Examination

Fixed ovary samples were sent to Hulk Bio-Tech Co., Ltd. (Hangzhou, China) for testing. Samples were dehydrated using a graded series of alcohol solutions (50%, 70%, 90%, 95%, and 100%), embedded with paraffin wax, and cut into 4 μm sections. Finally, the samples were stained with hematoxylin and eosin. A light microscope (Nikon camera, Tokyo, Japan) was used to observe the tissue sample and take pictures.

#### 2.3.3. Crude Protein Content and Amino Acid Composition Analysis in Hepatopancreas and Ovary

Using a protein determinator, the Dumas combustion method determined the crude protein content (FP-528, Leco, St. Joseph, MI, USA). For amino acid composition analysis, protein samples were hydrolyzed using 6 mol/L hydrogen chloride (HCl) at 110 °C for 24 h, and then HCl was removed with nitrogen. After that, the samples were redissolved in 0.1 mol/L HCl loading buffer and passed through a polyethersulfone ultrafiltration membrane with a pore size of 0.22 μm. Finally, an amino acid analyzer (L-8900, Hitachi, Tokyo, Japan) was used for determination according to the manufacturer’s instructions.

#### 2.3.4. Gene Expression Analysis by Real-Time Quantitative PCR (qPCR)

Table 3 listed the primers sequence of ovarian-development-related genes (*dmc1*, proliferating cell nuclear antigen, *pcna*; *vitellogenin*; drosophila steroid hormone, *cyp18a1*; ecdysone receptor, *ecr*; retinoid X receptor, *rxra*; vitellogenin1, *vg1*; vitellogenin receptor, *vgr*) and amino-acid-metabolism-related genes (target of rapamycin, *torc1*; 4E-binding protein, *4ebp*; eukaryotic translation initiation factor 4E, *eif4e*; ras homolog enriched in brain, *rheb*; ribosomal protein S6 kinase, *s6k*; protein kinase B, *akt*; regulatory-associated protein of mTOR, *raptor*; arginine transport vehicle, *cat-1*; nitric oxide synthesis, *nos*; cyclic guanosine monophosphate, *cgmp*). *β-actin* was used as an internal gene. All reagents used for qPCR were purchased from Vazyme Biotech Co., Ltd. (Nanjing, China). Total RNA from the samples was isolated using Total RNA Extraction Reagent. Subsequently, we evaluated the quality and quantity of the RNA samples. We used the Primer-Script^TM^ One-Step RT-PCR Kit from Vazyme Biotech Co., Ltd. to generate cDNA. We utilized the StepOne Plus Real-Time PCR system from Applied Biosystems and the SYBR Premix Ex Taq II kit from Vazyme Biotech Co., Ltd for qPCR. The reaction volume for amplification was 10 μL, comprising 5 μL of 2× SYBR Green I Master Mix, 0.2 μL each of forward and reverse gene-specific primers (0.2 μM), 4.2 μL of DEPC water, and 0.4 μL of cDNA. The PCR program consisted of an initial denaturation step at 95 °C for 2 min, followed by 45 cycles of 95 °C for 10 s, 58 °C for 10 s, and 72 °C for 20 s. The relative changes in the gene expression were analyzed by the 2^−ΔΔCt^ method.

### 2.4. Statistical Analysis

We conducted a statistical analysis using IBM^®^SPSS software version 26.0 (SPSS Inc., Chicago, IL, USA) with a significance value of *p* set at <0.05. Experiment 1 presented the quantitative data as mean ± standard error (SE) of three biological replicates (n = 3). We first conducted tests for normality and homoscedasticity to analyze the index values across different groups. If the statistical assumptions were met, we then used a one-way ANOVA to analyze the differences among the groups in the index values. Otherwise, the Kruskal–Wallis test was utilized. To determine the suitable regression model, orthogonal polynomial contrasts were utilized to examine whether the effect was linear, quadratic, and/or cubic. In Experiment 2, the independent-samples Student’s *t*-test was performed to evaluate the significance of differences between the control and the interference group.

## 3. Results

### 3.1. Experiment 1: 12-Week Arginine Feeding Experiment

#### 3.1.1. Statistics of Ovarian Development Stages and Ovary Histological Evaluation

**Figure 1:**
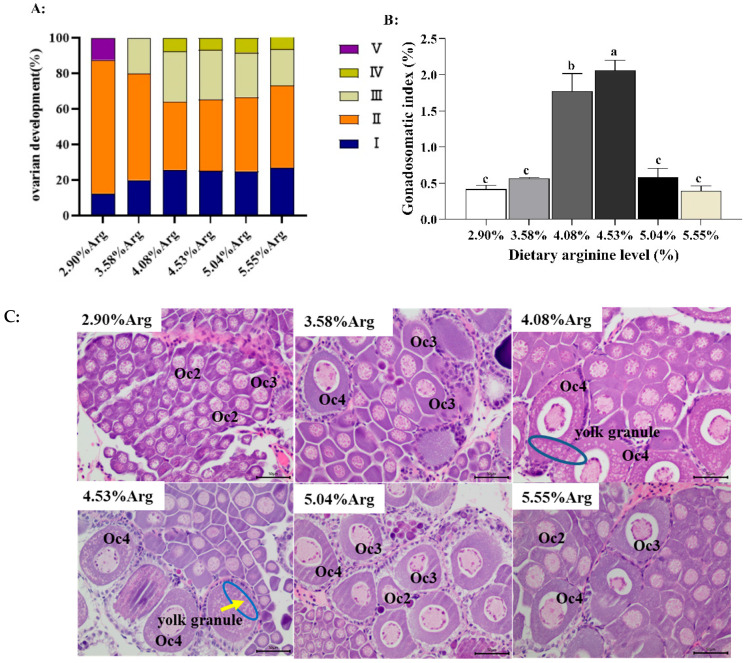
Dietary arginine supplementation could promote ovary development in *Litopenaeus vannamei*. (**A**) shows ovarian staging statistics; (**B**) shows the GIS (gonadosomatic index); (**C**) shows ovarian histology; a, b, c, and mean values of bars for the same parameter with different superscript letters were significantly different (*p* < 0.05). Data were reported as the means and SE of 3 replicates. Data with different superscripts significantly differed based on one-way ANOVA analysis (*p* < 0.05). Oc1, early previtellogenic oocyte; Oc2, late previtellogenic oocyte; Oc3, early vitellogenic oocyte; Oc4, late vitellogenic oocyte/mature oocyte; the yellow arrow indicates yolk granules, and the blue circle emphasizes the deposition density of yolk granules. The magnification was 400×, and the scale represents 50 µm.

Figure 1A shows the ovarian development stages and ovary histological evaluation of *L. vannamei* fed diets with different Arg levels (2.90%, 3.58%, 4.08%, 4.53%, 5.04%, and 5.55%). As shown, the stages of ovarian development were not synchronized, with II~V stages occurring simultaneously. Compared with the control group (2.90% Arg, without extra Arg supplementation), when 4.08% and 4.53% Arg were added to the diet, shrimp with stages III~V became relatively larger than the control group. Figure 1B shows when shrimp fed the 4.08% and 4.53% Arg diets, the gonadosomatic index achieved a higher value (*p* < 0.05) than the control.

As shown in Figure 1C, the abundance and diameter of oocytes were histologically assessed for classification to show the ovaries’ maturation degree. There was a statistically significant difference between the control and Arg supplementation groups. The control group contained mainly early yolk cells (Oc1), while the 4.08% and 4.53% Arg groups contained mainly late oocytes (Oc2) and mature oocytes (Oc4) filled with large deposits of yolk protein granules. The 3.58% and 5.55% Arg groups contained mainly early yolk cells (Oc3) and late yolk cells (Oc2). The 5.04% Arg group contained mainly early yolk cells (Oc3 and Oc1).

#### 3.1.2. Serum Hormone Concentrations Associated with Ovarian Development

**Figure 2:**
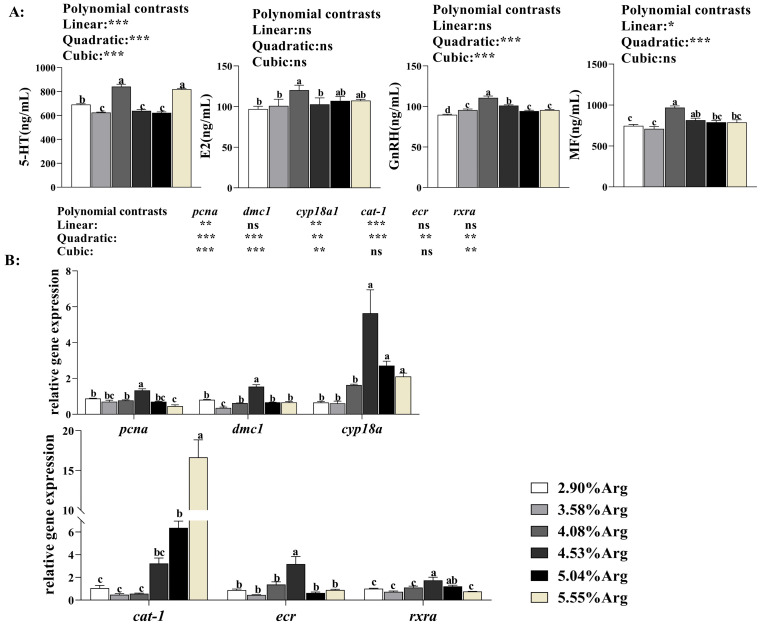
Dietary arginine supplementation could promote ovary development in *Litopenaeus vannamei*. (**A**) shows serum hormone concentrations associated with ovarian development; (**B**) shows the expression of ovarian development and arginine-transported genes in the ovarian tissue. ^a, b, c, d^, and mean values of bars for the same parameter with different superscript letters were significantly different (*p* < 0.05). Data were reported as the means and SE of 3 replicates. Data with different superscripts significantly differed based on one-way ANOVA analysis (*p* < 0.05). * means that the regression model was extremely and significantly suitable (*p* < 0.05), ** means that the regression model was extremely and significantly suitable (*p* < 0.01), *** means that the regression model was extremely and significantly suitable (*p* < 0.001), while ns means that the regression model was not suitable (*p* > 0.05) in the orthogonal polynomial contrasts analysis. 5-Hydroxytryptamine, 5-HT; gonadotropin-releasing hormone, GnRH; estradiol, E2; methylfarnesides, MF; proliferating cell nuclear antigen, *pcna*; ecdysone receptor, *ecr*; retinoid X receptor, *rxra*; arginine transport vehicle, *cat-1*; drosophila steroid hormone, *cyp18a*.

With increased Arg supplementation levels in the diets, as shown in Figure 2A, the serum concentrations of 5-HT, E2, GnRH, and MF showed a trend of first increasing and then decreasing. Shrimp fed with the 4.08% Arg levels, serum 5-HT, E2, GnRH, and MF concentrations achieved the highest value (*p* < 0.05) compared with the control group.

#### 3.1.3. Expression of Ovarian Development and Arg-Transported Genes in the Ovarian Tissue

With increased Arg supplementation levels in diets, as shown in Figure 2B, the expression levels of *pcna*, *dmc1*, *cyp18a*, *ecr,* and *rxra* in ovarian tissues showed a trend of first increasing and then decreasing. When the Arg supplementation level reached 4.08%, *pcna*, *cyp18a*, *ecr,* and *rxra* expression achieved the highest value (*p* < 0.05). The expression of *cat-1* showed a decreasing trend and then an increasing trend with the increased Arg levels (*p* < 0.05). When shrimp were fed with 4.53%, 5.04%, and 5.55% Arg, the *cat-1* expression was significantly (*p* < 0.05) higher compared with the control group.

#### 3.1.4. The Crude Protein Content and Amino Acid Composition of the Hepatopancreas and Ovary

**Figure 3:**
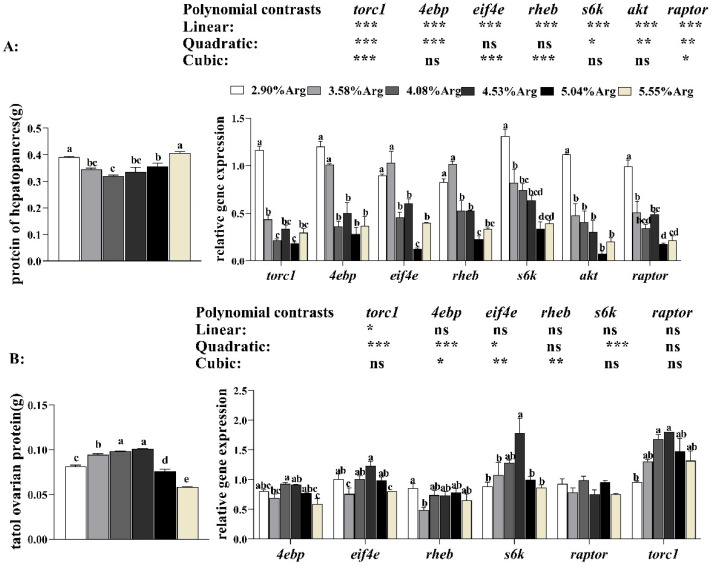
Dietary arginine supplementation could activate mTORC1-mediated protein metabolism in *Litopenaeus vannamei*. (**A**) shows the crude protein content and expression of genes related to the mTORC1 signaling pathway in the hepatopancreas; (**B**) shows the total ovarian protein and expression of genes related to the TOR pathway in the ovary. ^a, b, c, d^, and mean values of bars for the same parameter with different superscript letters were significantly different (*p* < 0.05). Data were reported as the means and SE of 3 replicates. Data with different superscripts significantly differed based on one-way ANOVA analysis (*p* < 0.05). * means that the regression model was extremely and significantly suitable (*p* < 0.05), ** means that the regression model was extremely and significantly suitable (*p* < 0.01), *** means that the regression model was extremely and significantly suitable (*p* < 0.001), while ns means that the regression model was not suitable (*p* > 0.05) in the orthogonal polynomial contrasts analysis. Protein kinase B, *akt*; ras homologue enriched in brain, *rheb*; target of rapamycin, *torc1*; regulatory-associated protein of mTOR, *raptor*; ribosomal protein S6 kinase, *s6k*; eukaryotic translation initiation factor 4E, *eif4e*; 4E-binding protein, *4e-bp*.

With increased Arg supplementation levels in the diets, as shown in Figure 3A, the crude protein content in hepatopancreas decreased and then increased. When the Arg supplementation level reached 4.08%, the crude protein content was the lowest and significantly lower (*p* < 0.05) than that in the control group. In contrast, with the increase in Arg levels, the total protein content in the ovary showed a trend of first increasing and then decreasing. When 4.53% Arg was added, the crude protein content was the highest and significantly higher (*p* < 0.05) than the control group.

With increased Arg supplementation levels in the diets, the total essential and non-essential amino acid content showed increasing and decreasing trends in the hepatopancreas and ovary (Supplementary Figure S1). Shrimp fed with a 4.53% Arg supplementation level achieved the highest value (*p* < 0.05) of the content of total essential and non-essential amino acids in the hepatopancreas and ovary. The Arg content showed an increasing and decreasing trend in the hepatopancreas and ovary, and shrimp fed with 4.53% Arg achieved the highest value (*p* < 0.05).

#### 3.1.5. Gene Expression Related to mTORC1 Signaling Pathway in the Ovary and Hepatopancreas

Gene expression related to the mTORC1 signaling pathway showed tissue specificity. In the hepatopancreas (Figure 3A), the expression levels of *torc1*, *4ebp*, *s6k*, *akt,* and *raptor* showed a downward trend with increased Arg supplementation levels (*p* < 0.05). Compared with the control group, shrimp fed with 3.58%, 4.08%, 4.53%, 5.04%, and 5.55% Arg had lower expression levels (*p* < 0.05) of torc1, akt, and *raptor.* In the ovary, the expression levels of *eif4e* and *s6k* showed an upward and then downward trend. In shrimp fed with 4.53% Arg, the eif4e, s6k, and torc1 expression levels reached the highest value and were significantly higher (*p* < 0.05) than the control group. There was no significant difference in the raptor expression level (*p* > 0.05). The *4ebp* expression level was higher but had no significance in the 4.08% and 4.53% groups compared to the control group.

#### 3.1.6. Expression of Vitellogenin and Vgr Genes in the Ovary and Hepatopancreas

**Figure 4:**
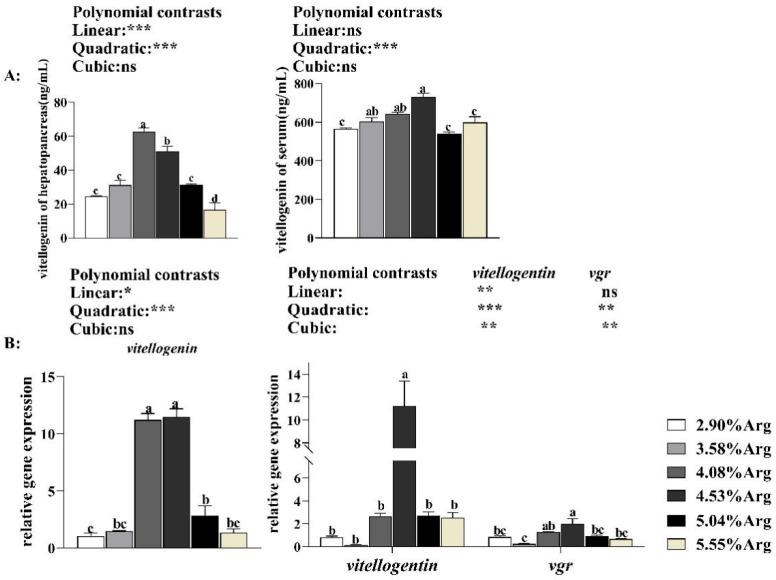
Dietary arginine supplementation could enhance endogenous and exogenous vitellogenin synthesis in *Litopenaeus vannamei*. (**A**) shows the vitellogenin contents in the hepatopancreas serum; (**B**) shows the expression of *vitellogenin* (hepatopancreas and ovary) and ovary *vgr*. ^a, b, c, d^, and mean values of bars for the same parameter with different superscript letters were significantly different (*p* < 0.05). Data were reported as the means and SE of 3 replicates. Data with different superscripts significantly differed based on one-way ANOVA analysis (*p* < 0.05). * means that the regression model was extremely and significantly suitable (*p* < 0.05), ** means that the regression model was extremely and significantly suitable (*p* < 0.01), and *** means that the regression model was highly significant suitable (*p* < 0.001). In contrast, ns means that the regression model was unsuitable (*p* > 0.05) in the orthogonal polynomial contrasts analysis. Vitellogenin receptor, *vgr*.

With increased Arg supplementation levels in the diets, as shown in Figure 4B, the expression of *vitellogenin* and *vgr* showed an upward and downward trend in the ovary and hepatopancreas. When the Arg supplementation level reached 4.53% vitellogenin, *the vgr expression levels* achieved the highest value and were significantly higher (*p* < 0.05) than the control group. Compared with the control group, shrimp fed with 4.08%, 4.53%, and 5.04% Arg significantly increased (*p* < 0.05) the vitellogenin expression level in the hepatopancreas. Shrimp fed with 4.53% Arg significantly increased (*p* < 0.05) the ovary’s vitellogenin and vgr expression levels.

#### 3.1.7. Vitellogenin Concentration in Serum and Hepatopancreas

With increased Arg supplementation levels in the diets, as shown in Figure 4A, the serum vitellogenin concentration showed an upward and downward trend. Shrimp fed with 4.53% Arg achieved the highest concentration (*p* < 0.05) than the control group. With the increasing Arg supplementation, the hepatopancreas vitellogenin concentration showed an upward and downward trend, and shrimp fed with 4.08% Arg achieved the highest value. Compared with the control group, shrimp fed with 4.08% and 4.53% Arg significantly increased (*p* < 0.05) the hepatopancreas and serum vitellogenin concentration.

#### 3.1.8. Arg Metabolism Associated Enzyme Activity and Gene Expression

**Figure 5:**
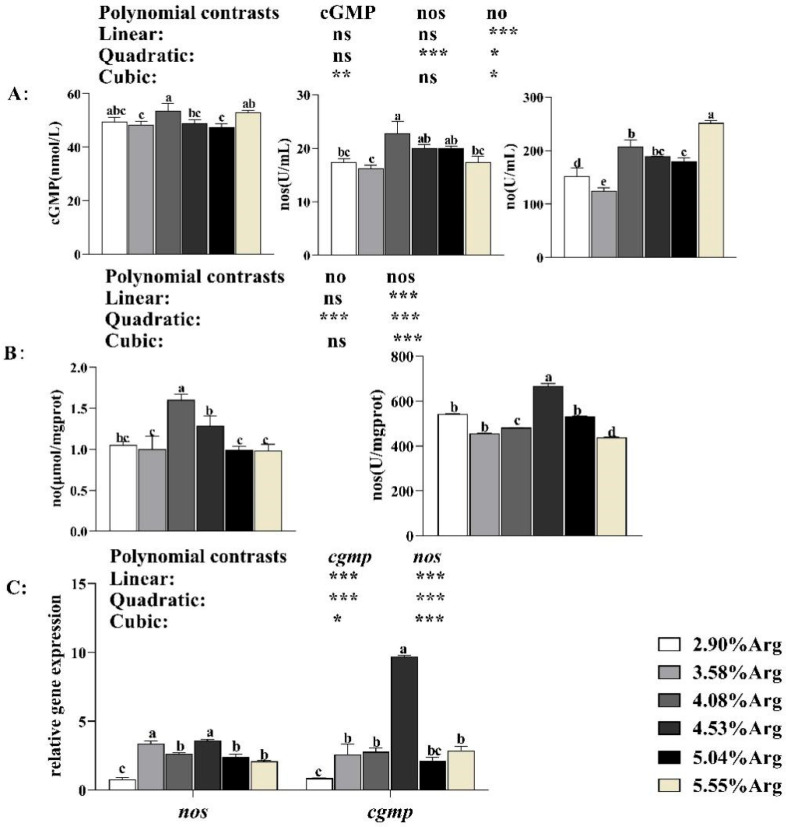
Dietary arginine supplementation could activate NO-sGC-cGMP signaling ways in *Litopenaeus vannamei*. (**A**) shows arginine metabolism enzyme activity in serum; (**B**) shows arginine metabolism enzyme activity in hepatopancreas; (**C**) shows the expression of genes related to arginine metabolism in the ovary. ^a, b, c, d, e^, and mean values of bars for the same parameter with different superscript letters were significantly different (*p* < 0.05). Data with different superscripts significantly differed based on one-way ANOVA analysis (*p* < 0.05). * means that the regression model was extremely and significantly suitable (*p* < 0.05), ** means that the regression model was extremely and significantly suitable (*p* < 0.01), *** means that the regression model was extremely and significantly suitable (*p* < 0.001), while ns means that the regression model was not suitable (*p* > 0.05) in the orthogonal polynomial contrasts analysis. Data are reported as the means and SE of 3 replicates. Nitric oxide synthesis, *nos*; cyclic guanosine monophosphate, *cgmp*.

As shown in Figure 5A, the serum NOS and cGMP concentration showed an upward and downward trend. When the Arg supplementation level reached 4.53%, the NOS and cGMP concentrations achieved the highest value in serum. The serum NO concentration increased (*p* < 0.05) when shrimp were fed with 4.08%, 4.53%, 5.04%, and 5.55% Arg. In the hepatopancreas (Figure 4B), when the Arg supplementation level reached 4.08%, the NO concentration achieved the highest value and was significantly higher (*p* < 0.05) than the control. When the Arg supplementation level reached 4.53%, the NOS concentration achieved the highest value and was significantly higher (*p* < 0.05) than the control. As shown in Figure 4C, the expression levels of *nos* and *cgmp* in the ovary showed an increasing and decreasing trend. When the Arg supplementation level reached 4.053%, the expression levels of *nos* and *cgmp* in the ovary achieved the highest value and were significantly higher (*p* < 0.05) than the control.

### 3.2. Experiment 2 RNA Interference Experiment for Silencing TOR and NOS Genes

#### 3.2.1. TOR Knockdown Experiment

**Figure 6:**
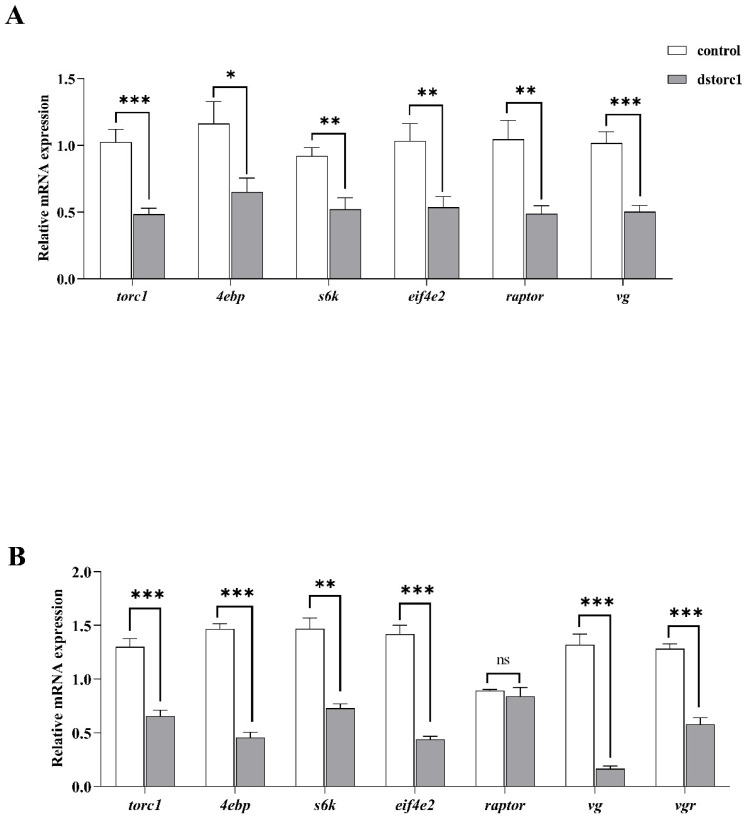
The expression of the genes related to the mTOR1 pathway, *vg,* and *vgr* after injecting the dsTOR in *Litopenaeus vannamei*. (**A**) The expression of genes related to the mTOR1 pathway and *vg* in hepatopancreas; (**B**) the expression of the genes related to the mTOR1 pathway, *vg*, and *vgr* in ovary. Data with different superscripts significantly differed based on the independent samples Student’s *t*-test (*p* < 0.05). * means that the regression model was extremely and significantly suitable (*p* < 0.05), ** means that the regression model was extremely and significantly suitable (*p* < 0.01), *** means that the regression model was extremely and significantly suitable (*p* < 0.001), while ns means that the regression model was not suitable (*p* > 0.05). Data are reported as the means and SE of 3 replicates.

As shown in Figure 6, 48 h after the dsTOR injection, the expression levels of hepatopancreas *torc1*, *4ebp*, *s6k*, *eif4e2*, *vg*, and raptor in the dsTOR group were downregulated (*p* < 0.05) significantly compared to the control group. The expression of ovary *torc1*, *4ebp*, *s6k*, *eif4e2*, *vg*, and *vgr* in the dsTOR group was downregulated (*p* < 0.05) compared to the control group.

#### 3.2.2. NOS Knockdown Experiment

**Figure 7:**
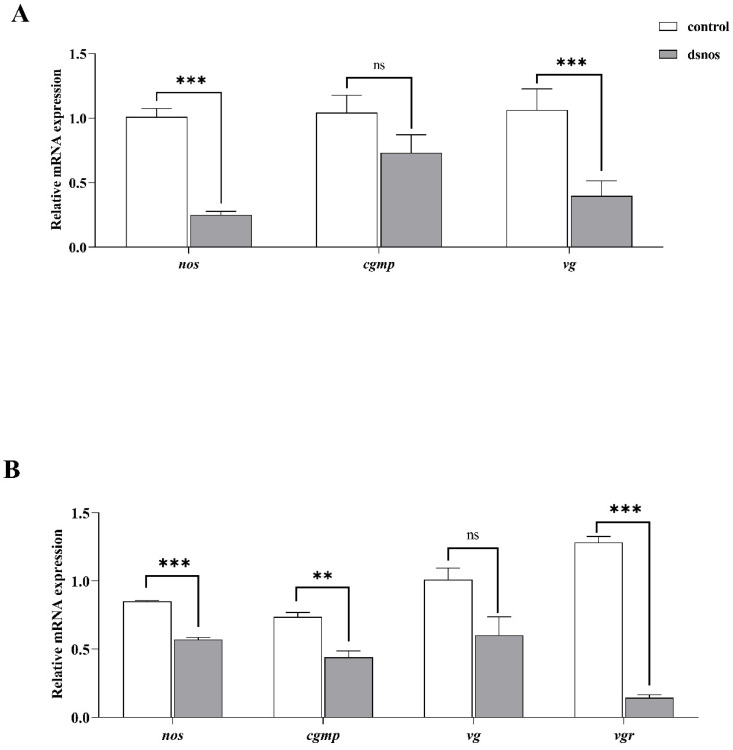
The expression of the genes related to the NO-sGC-cGMP pathway, *vg*, and *vgr* after injecting the dsNOS in *Litopenaeus vannamei*. (**A**) The expression of *nos*, *cgmp,* and *vg* in hepatopancreas; (**B**) the expression of the genes (*nos*, *cgmp*, *vg,* and *vgr*) in the ovary. Data with different superscripts significantly differed based on the independent samples Student’s *t*-test (*p* < 0.05). ** means that the regression model was extremely and significantly suitable (*p* < 0.01), *** that means the regression model was extremely and significantly suitable (*p* < 0.001), while ns means that the regression model was not suitable (*p* > 0.05). Data are reported as the means and SE of 3 replicates.

As shown in Figure 7, 48 h after the dsNOS injection, the expression levels of hepatopancreas *nos*, *cgmp,* and *vg* were downregulated (*p* < 0.05) compared to the control group. The expression levels of ovary *nos*, *cgmp,* and *vgr* were downregulated (*p* < 0.05) significantly compared to the control group.

## 4. Discussion

Currently, the literature on the regulatory role of Arg in ovary development is very limited in both aquatic animals and mammals. In aquatic animals, only two reports have outlined the promoting effect of Arg on ovary development in Chinese mitten crab and Nile tilapia, respectively [7,8]. Our data also suggested that dietary Arg supplementation could improve ovarian growth and development in *L. vannamei* after the 12-week raring cycle. When 4.08% and 4.53% Arg were added to the diet, the numbers of shrimp with stages III~V reached relatively high, and the gonadosomatic index achieved a higher value than the control. Thus, Arg had a dose effect on ovarian development regulation. The higher the added dose of Arg, the more absorbed (identified by increased Arg transporter *cat-1* with increased Arg supplementation level in diets), which does not necessarily effectively promote ovary development.

Ovarian development is inextricably linked and controlled by neurotransmitters and steroid hormones in shrimp, which act in combination by stimulating neuropeptide secretion to promote ovarian maturation. Emerging evidence suggested that 5-HT, GnRH, MF, and E2 have been shown to shorten the duration of ovarian maturation and spawning in shrimp and crab species through in vivo injection [16,17,18,19]. However, the relationship between nutrition, hormone function, and control (endocrinology) remains one of the least-known areas of crustacean and shrimp biology. In our study, we found that appropriate Arg supplementation could increase the serum 5-HT, E2, GnRH, and MF concentration, which explained the promoting effect of Arg on ovary development in *L. vannamei*. In addition, our study further examined the expression levels of genes related to ovarian development. The result showed that appropriate Arg supplementation upregulated the expression of *pcna*, *dmc1*, *cyp18a*, *ecr,* and *rxra* in ovarian tissues of *L. vannamei*. *mjPCNA* is a crucial player in the development of testes and ovaries in *Marsupenaeus japonicus*, primarily responsible for regulating mitosis and meiosis [20]. *LvDmc1* is a specific marker for the development of germ cells, as it is expressed solely in premeiotic oogonia/spermatogonia [21]. Cyp18a1 plays a critical role in the breakdown of 20-E and inhibiting ecdysteroidogenesis in insects, as it is the primary enzyme responsible for these processes [22]. The ecdysone signaling pathway involves the initial binding of active 20-E to its *ecr*, followed by forming a heterodimer complex with another nuclear receptor RXR. The *ecr* expression level significantly increased in the Oziothelphusa senex and S. paramamosain ovaries during the vitellogenic stage [23,24].

During ovarian development in crustaceans, many nutrients synthesized by the hepatopancreas are transported to the developing oocytes, especially lecithin (Vn) and lipids. Vitellin (Vn) is the main component of mature oocytes, accounting for 60–90% of the total ovarian protein of shrimp. Structurally, Vn is an apolipoprotein that combines with lipids to form a lipoprotein encoded by its precursor gene vitellogenin (a large molecular lipoprotein with a molecular weight of 290–700 kDa), which is processed into a mature protein by proteolytic cleavage after translation. The vitellogenin levels in hemolymph are used as an indicator of female reproductive status in crustaceans. Through yolk deposition, protein and lipid substances are transported to the developing ovaries during vitellogenesis. Vitellogenesis in crustaceans is an energy-consuming process, and it is generally believed that it is carried out in two ways, either endogenous or exogenous. The hepatopancreas is the major exogenous vitellogenin synthesis and uptake site for the ovarian maturation of *L. vannamei*. At the same time, the ovary is the endogenous vitellogenin synthesis and uptake site for the ovarian maturation of *L. vannamei* [25]. In our study, shrimp fed with 4.08% and 4.53% Arg had higher serum and hepatopancreas vitellogenin concentrations, and hepatopancreas increased the *vitellogenin* expression level. Meanwhile, the expression of *vgr* in the ovary was significantly upregulated when shrimp were fed with 4.08% and 4.53% Arg. In shrimp, extra ovarian Vg synthesized in the hepatopancreas is secreted into the hemolymph and sequestered into developing oocytes by receptor-mediated endocytosis by VGR [26]. These results indicated that 4.08% and 4.53% Arg supplementation promoted the hepatopancreas’ exogenous vitellogenin synthesis and was transported into the ovary through VGR, further promoting ovarian maturation in *L. vannamei.* Furthermore, when shrimp were fed with 4.53% Arg, the vitellogenin expression achieved a higher value in the ovary, indicating endogenous vitellogenin synthesis in ovarian maturation in *L. vannamei.* Consistent with this result, the ovarian histological examination showed that when 4.08% and 4.53% Arg were fed, late oocytes and mature oocytes were filled with large deposits of yolk protein granules in the ovary. Therefore, dietary Arg supplementation could promote ovary development through endogenous and exogenous vitellogenin synthesis in *L. vannamei.*

The mTORC1 signaling pathway controls many metabolic processes and is regulated by amino acid signals, especially Arg [27]. We determined the gene expression levels of two components (*tor* and *raptor*), two upstream (*akt* and *rheb*) and three downstream (*s6k*, *eif4e,* and *4e-bp*), of the mTORC1 signaling pathways. In our study, the crude protein contents in the ovary showed increasing and decreasing trends, and the gene levels of the mTORC1-mediated signaling pathways showed the same trend. Interestingly, when the dietary Arg increased, the hepatopancreas crude protein contents showed a generally decreasing trend, and the expression levels of genes related to the mTORC1 pathway were significantly downregulated in hepatopancreas in general. However, vitellogenin expression showed an increasing and then decreasing trend. In shrimp, the hepatopancreas is the main organ of energy metabolism, especially protein synthesis required for the growth of organisms. It might be suspected that amino acids were mainly used to synthesize vitellogenin, inhibiting other proteins’ synthesis.

Oocyte maturation is essential for reproduction through sexual reproduction in insects, crustaceans, and other oviparous animals. In recent years, the role of cGMP signaling in oocyte maturation has received much attention in mammals. Studies have shown that the NO system is involved in the maturation of animal oocytes through the regulation of cGMP oocyte maturation in animals. NO is an inorganic radical gas that binds to sGC, catalyzing the conversion of GTP to cGMP [28]. NO can act as an intracellular biological messenger, freely crossing the cell membrane to regulate the physiological functions of various cells in the ovary in a relatively specific manner. Studies have shown that the NO-sGC-cGMP signaling pathway plays an important role in steroid hormone synthesis (estradiol, progesterone, etc.), folliculogenesis, and oocyte maturation in vitro and in vivo mammalian models [29]. Li et al. (2018) used zebrafish as a model to demonstrate for the first time in fish the involvement of NO-sGC-cGMP in oocyte maturation [30]. As a functional amino acid, Arg is a basic component of protein and an important substrate for nitric oxide synthesis. Arg is the only substrate used for NO synthesis and is involved in reproductive physiology through the Arg-NO pathway, regulating follicular development, steroid hormone synthesis, and ovulation. Studies have shown that NOS metabolizes L-Arg to produce NO and L-guanine, which diffuse freely across the cell membrane and induce reactions in adjacent cells [31]. NOS is the primary rate-limiting factor for NO production, and various isozymes of NOS are expressed and localized in the ovaries of different species [32]. NOS, which is highly homologous to insect NOS gene sequences, was identified in land crabs. NOS mRNA expression levels were significantly increased in the ovary, testis, and oculo-palpebral ganglion [22]. The addition of Arg to mouse ovarian tissue cultures has been reported to accelerate the maturation of primordial follicles [33]. Arg feeding increased the expression of eNOS and sGC proteins and mRNA in sheep oocytes and accelerated cell proliferation [34]. Arg injection activated the NO-sGC-cGMP signaling pathway to promote follicle development and alleviate malnutrition-induced delayed estrus in ewes. Seven hours of incubation with Arg significantly increased cGMP levels and the proportion of bovine oocyte nuclei entering maturation [35]. Our study found that dietary Arg supplementation could increase serum no, nos, and cgmp while upregulating the gene expression levels of *nos* and *cgmp* in the ovary and hepatopancreas in *L. vannamei.* Therefore, we hypothesized that Arg could promote ovary development through the NO-sGC-cGMP signaling pathway in *L. vannamei.*

We successfully built NOS and TOR knockdown models to further elucidate whether the NO-sGC-cGMP and mTORC1 signaling pathways that mediated the Arg regulate ovary development in *L. vannamei.* We observed that when the NO-sGC-cGMP and mTORC1 signaling pathways were inhibited, the *vgr* and *vg* gene expression was downregulated in *L. vannamei.* This result confirmed that the NO-sGC-cGMP and mTORC1 signaling pathways that mediated the Arg regulated ovary development in *L. vannamei.*

## 5. Conclusions

Overall, dietary supplementation of 4.08–4.53% Arg enhanced endogenous and exogenous vitellogenin synthesis to promote ovarian development in *L. vannamei*. The NO-sGC-cGMP signaling pathway and mTORC1 pathway mediated Arg regulating the ovarian development of *L. vannamei*. The results of our study could potentially offer valuable theoretical backing and serve as a useful reference for shrimp reproduction improvement through artificial broodstock diets.

## Figures and Tables

**Table 1:** Ingredient content and composition of the arginine diets (air-dry basis, %).

Ingredients (g/100 g)	2.90 %Arg	3.58 %Arg	4.08 %Arg	4.53 %Arg	5.04 %Arg	5.55 %Arg
Fish meal (Peru)	35.00	35.00	35.00	35.00	35.00	35.00
Soy protein concentrate	5.00	5.00	5.00	5.00	5.00	5.00
Soybean meal	4.00	4.00	4.00	4.00	4.00	4.00
Krill meal	9.00	9.00	9.00	9.00	9.00	9.00
Baker’s yeast hydrolysate	5.00	5.00	5.00	5.00	5.00	5.00
Domestic fish meal	2.00	2.00	2.00	2.00	2.00	2.00
Squid offal powder	2.50	2.50	2.50	2.50	2.50	2.50
Squid powder	2.50	2.50	2.50	2.50	2.50	2.50
Sandworms	1.00	1.00	1.00	1.00	1.00	1.00
Wheat flour	22.19	22.19	22.19	22.19	22.19	22.19
Fish oil	1.00	1.00	1.00	1.00	1.00	1.00
Soy lecithin	1.50	1.50	1.50	1.50	1.50	1.50
Cholesterol	0.50	0.50	0.50	0.50	0.50	0.50
Mineral premix ^1^	1.00	1.00	1.00	1.00	1.00	1.00
Vitamin premix ^2^	0.50	0.50	0.50	0.50	0.50	0.50
Ca (H_2_PO4)_2_	1.50	1.50	1.50	1.50	1.50	1.50
Choline chloride	0.30	0.30	0.30	0.30	0.30	0.30
Non-essential amino acid premix ^3^	5.51	4.41	3.31	2.20	1.10	0.00
Arginine	0.00	0.50	1.00	1.50	2.00	2.50
Cellulose	0.00	0.60	1.20	1.81	2.41	3.01
Total	100.00	100.00	100.00	100.00	100.00	100.00
Crude nutrient levels ^4^						
Protein (%)	54.00	54.38	54.71	55.25	54.07	54.00
Crude lipid (%)	9.62	9.54	9.66	9.56	9.53	9.60
Moisture (%)	8.48	9.00	8.76	8.32	8.88	8.76
Ash (%)	10.95	10.91	11.27	10.75	11.48	10.96
Arginine (%)	2.90	3.58	4.08	4.53	5.04	5.55

Mineral premix ^1^: 3.51 g/kg Fe as C_6_H_5_O_7_ Fe·5H_2_O; 6.62 g/kg Cu as CuSO_4_·5H_2_O; 0.01 g/kg I as KIO_3_; 238.96 g/kg Mg as MgSO_4_·7H_2_O; 298.58 g/kg K as K_2_SO_4_; 49.21 g/kg Na as NaCl; 34.01 g/kg Ca as C_6_H_10_O_6_Ca.5H_2_O; 1.60 g/kg Co as CoSO_4_·7H_2_O; 10.31 g/kg Zn as ZnSO_4_.7H_2_O; 0.25 g/kg MnSO_4_·H_2_O. Vitamin premix ^2^: 2,500,000 IU vitamin A; 500,000 IU vitamin D_3_; 25,000 IU vitamin E (DL-α tocopherol acetate); 5.63 g/kg vitamin K_3_; 11.25 g/kg vitamin B1; 9.5 g/kg vitamin B_2_; 10 g/kg vitamin B_6_; 0.02 g/kg vitamin B_12_; 2 g/kg folic acid; 0.375 g/kg biotin; 37.5 g/kg nicotinamide; 21.5 g/kg D-pantothenic acid; 95 g/kg vitamin C; 80 g/kg inositol. Non-essential amino acid premix ^3^ was aspartic acid and glycine (supplementation ratio = 1:1). Nutrient levels ^4^ were measured values (dry matter basis).

**Table 2:** Primers for RNA interference experiment.

Primers	Sequence (5′-3′)	TM
T7-dsGFP-F	TAATACGACTCACTATAGGGCGACGTAAACGGCCACAAGT	70.31
T7-dsGFP-R	TAATACGACTCACTATAGGGCTTGTACAGCTCGTCCATGC	70.61
T7-dsTOR-F	TAATACGACTCACTATAGGGGTTATGTCACCACGGAGTT	67.43
T7-dsTOR-R	TAATACGACTCACTATAGGGAGTGCCACCCGAAGA	68.52
T7-dsNOS-F	TAATACGACTCACTATAGGGTCACGCCCGTATTCCA	67.76
T7-dsNOS-R	TAATACGACTCACTATAGGGCGTTCCGCTAACTTTCAT	68.32
*tor* for qPCR	F: TTTGAAGTTGCCATGACCCG	52.52
*tor* for qPCR	R: GAGACGCCAATTCAGCAGAG	54.62
*nos* for qPCR	F: ATGAGGACGGGACCATCATC	53.12
*nos* for qPCR	R: TTGTATTCGGGGTGGCTGAT	54.91

**Table 3:** Specific primers for real-time quantitative PCR.

Gene	Accession No.	Primers (5′-3′)
*dmc1*	HQ116385.1	F: CGAGGAATACAACGTGTCTGTC R: ATTCGGGGCTGTCGTAGAT
*pcna*	JN034913.1	F: ATTGCCTTCTGGGGAGTTC R: CAAGCAAAGGTGAGCGTGA
*torc1*	XM_027372359.1	F: TGCCAACGGGTGGTAGA R: GGGTGTTTGTGGACGGA
*vitellogenin*	KM077131.1	F: GAACCCTAAGGCTATCATCACTG R: AGGTCGCTCTTCCATCTTTACT
*cyp18a1*	XM_027360159.1	F: CTCCTGAGGTGCCTGTCG R: GGGATGTAGTTGGCGATG
*ecr*	XM_027356283.1	F: GTTATGACGCAAAGACCAAT R: TTACGACAGAAGCGAAAGAG
*rxra*	Reference [13]	F: CCCTCTCCTCTCTCCCTC R: GGTTGCTGCTCCCATTAC
*4ebp*	XM_027367939.1	F: ATGTCTGCTTCGCCCGTCGCTCGCC R: GGTTCTTGGGTGGGCTCTT
*vgr*	MN807241.1	F: TCTCCTCGTCTTGGCTCT R: GCAAACTGCGGCGGCTGG
*eif4e*	XM_027354395.1	F: TGGAATCAAACCTATGTGGG R: GTCCTCCTGGAAGCGTA
*cat-1*		F: CCTGTCTCCAGCCTGTTTGAAGTTG R: CCAGTCAATGCTCCCAAATGTGCTC
*vg1*	MN105878.1	F: GCCGTAGAAGCCAAGGTA R: AAGGCAGTGAAAGGAGCA
*β-actin* *akt* *rheb* *s6k* *raptor* *nos* *cgmp*	Reference [14] XM_027364781.1 MG696863.1 XM_027368997.1 XM_027360909.1 Reference [15] XM_027376715.1	F: CCTCGGTTCTATTTTGTCGGTTT R: GCAGATGCTTTCGCAGTAGGT F: AAATGACTATGGACGAGGTGTT R: GTTGATGGTGATGTAGAAGGGG F: AGGAAAGTGGCCGTTATGGG R: TACCAGCTCCAGGCCATACT F: GCAAGAGGAAGACGCCATA R: CCGCCCTTGCCCAAAACCT F: CTGCTTTCCAGGCTACTC R: TCACAATCCAAGGTCCAG F: GAGCAAGTTATTCGGCAAGGC R: TCTCTCCCAGTTTCTTGGCGT F: TTTTACAACCCCCACCCCAC R: AGAGAGAGAGGAGGGGCAAG

Proliferating cell nuclear antigen, *pcna*; ecdysone receptor, *ecr*; retinoid X receptor, *rxra*; arginine transport vehicle, *cat-1*; drosophila steroid hormone, *cyp18a*; protein kinase B, *akt*; ras homolog enriched in brain, *rheb*; target of rapamycin complex 1, *torc1*; regulatory-associated protein of mTOR, *raptor*; ribosomal protein S6 kinase, *s6k*; eukaryotic translation initiation factor 4E, *eif4e*; 4E-binding protein, *4e-bp*; vitellogenin receptor, *vgr*; nitric oxide synthesis, *nos*; cyclic guanosine monophosphate, *cgmp*.

## Data Availability

The datasets analyzed during the current study are available from the corresponding author upon reasonable request.

## Supplementary Materials

The following supporting information can be downloaded at: https://www.mdpi.com/article/10.3390/ani14131986/s1, Table S1. Effects of dietary arginine supplementation ovary amino acids composition in *Litopenaeus vannamei*. Table S2. Effects of dietary arginine supplementation hepatopancreas amino acids composition in *Litopenaeus vannamei.* Figure S1. The result of dsGFP, dsTORC1 and dsNOS.

## Abbreviations

*Litopenaeus vannamei*, *L. vannamei;* arginine, Arg; polymerase chain reaction, PCR; double-stranded RNA (dsRNAs); nitric oxide synthase, NOS; target of rapamycin, TOR; nitric oxide, NO; anti-Mullerian hormone, AMH; progesterone, PROG; 5-hydroxytryptamine, 5-HT; gonadotropin-releasing hormone, GnRH; estradiol, E2; vitellogenin, VTG; methylfarnesides, MF; cyclic guanosine monophosphate, cGMP; proliferating cell nuclear antigen, *pcna*; ecdysone receptor, *ecr*; retinoid X receptor, *rxra*; arginine transport vehicle, *cat-1*; drosophila steroid hormone, *cyp18a*; protein kinase B, *akt*; ras homologue enriched in brain, *rheb*; target of rapamycin complex 1, *torc1*; regulatory-associated protein of mTOR, *raptor*; ribosomal protein S6 kinase, *s6k*; eukaryotic translation initiation factor 4E, *eif4e*; 4E-binding protein, *4e-bp*; vitellogenin receptor, *vgr*; nitric oxide synthesis, *nos*; cyclic guanosine monophosphate, *cgmp*; early previtellogenic oocyte, Oc1; late previtellogenic oocyte, Oc2; early vitellogenic oocyte, Oc3; late vitellogenic oocyte/mature oocyte, Oc4.

## References

[1] FAO (2020). World Food Situation. http://www.fao.org/worldfoodsituation/csdb/en/.

[2] Yang D.Z., Wang C.J., Kou N., Xing J.B., Li X., Zhao H., Luo M. (2022). Gonadal maturation in *Litopenaeus vannamei* fed on four different polychaetes. Aquacult. Rep..

[3] Wouters R., Lavens P., Nieto J., Sorgeloos P. (2001). Penaeid shrimp broodstock nutrition: An updated review on research and development. Aquaculture.

[4] Gao H. (2020). Amino acids in reproductive nutrition and health. Adv. Exp. Med. Biol..

[5] Zhang G.M., Guo Y.X., Cheng C.Y., El-Samahy M.A., Tong R., Gao X.X., Deng K.P., Wang F., Lei Z.H. (2020). Arginine infusion rescues ovarian follicular development in feed-restricted Hu sheep during the luteal phase. Theriogenology.

[6] Uyanga V.A., Xin Q., Sun M.F., Zhao J.P., Wang X.J., Jiao H.C., Onagbesan O.M., Lin H. (2022). Research Note: Effects of dietary L-arginine on the production performance and gene expression of reproductive hormones in laying hens fed low crude protein diets. Poult. Sci..

[7] Wassef E.A., El-Husseiny O.M., El-Kasheif M.A., Aboseif A.M., Suloma A. (2017). Effect of different dietary lipid sources and arginine supplementation on body-composition and gonadal development of young Nile tilapia (*Oreochromis niloticus*). J. Egypt. Acad. Soc. Environ. Develop..

[8] Qi C., Wang X., Han F., Jia Y., Lin Z., Wang C., Lu J., Yang L., Wang X., Li E. (2019). Arginine supplementation improves growth, antioxidant capacity, immunity and disease resistance of juvenile *Chinese mitten crab*, *Eriocheir sinensis*. Fish Shellfish Immunol..

[9] Zhou Q.C., Zeng W.P., Wang H.L., Wang T., Wang Y.L., Xie F.J. (2012). Dietary arginine requirement of juvenile Pacific white shrimp, *Litopenaeus vannamei*. Aquaculture.

[10] Palma J., Andrade J.P., Lemme A., Bureau D.P. (2015). Quantitative dietary requirement of juvenile Atlantic ditch shrimp Palaemonetes varians for lysine, methionine and arginine. Aquac. Res..

[11] Ngernsoungnern A., Ngernsoungnern P., Weerachatyanukul W., Chavadej J., Sobhon P., Sretarugsa P.J.A. (2008). The existence of gonadotropin-releasing hormone (GnRH) immunoreactivity in the ovary and the effects of GnRHs on the ovarian maturation in the black tiger shrimp *Penaeus monodon*. Aquaculture.

[12] Tinikul Y., Poljaroen J., Tinikul R., Anuracpreeda P., Chotwiwatthanakun C., Senin N., Poomtong T., Hanna P.J., Sobhon P.J.A. (2014). Effects of gonadotropin-releasing hormones and dopamine on ovarian maturation in the Pacific white shrimp, *Litopenaeus vannamei*, and their presence in the ovary during ovarian development. Aquaculture.

[13] Liu J., Zhou T., Wang C., Chan S., Wang W. (2021). Deciphering the molecular regulatory mechanism orchestrating ovary development of the Pacific whiteleg shrimp *Litopenaeus vannamei* through integrated transcriptomic analysis of reproduction-related organs. Aquaculture.

[14] Chen X., Yang H., Ruan Y., Zhou M., Liu J., Li Z., Wu X., Ren C., Zhang X., Zhang J. (2023). Pacific white shrimp (*Litopenaeus vannamei*) vitelline membrane outer layer protein 1 (VMO1) is produced in the hepatopancreas and transported into ovarian oocytes during vitellogenesis. Gene.

[15] Yao C.L., Ji P.F., Wang Z.Y., Li F.H., Xiang J.H. (2010). Molecular cloning and expression of NOS in shrimp, *Litopenaeus vannamei*. Fish Shellfish Immunol..

[16] Tinikul Y., Poljaroen J., Nuurai P., Anuracpreeda P., Chotwiwatthanakun C., Phoungpetchara I., Kornthong N., Poomtong T., Hanna P.J., Sobhon P. (2011). Existence and distribution of gonadotropin-releasing hormone-like peptides in the central nervous system and ovary of the *Pacific white shrimp*, *Litopenaeus vannamei*. Cell Tissue Res..

[17] Swetha C.H., Girish B.P., Reddy P.S. (2016). Elucidation of the role of estradiol and progesterone in regulating reproduction in the edible crab, Oziothelphusa senex senex. RSC Adv..

[18] Jayasankar V., Tomy S., Wilder M.N. (2020). Insights on molecular mechanisms of ovarian development in decapod crustacea: Focus on vitellogenesis-stimulating factors and pathways. Front. Endocrinol..

[19] Jin S.B., Yue D., Fu H.T., Jiang S.F., Xiong Y.W., Qiao H., Zhang W.Y., Gong Y.S., Wu Y. (2022). Effects of dietary supplementation with 17 beta-estradiol and 17 alpha-methyltestosterone on growth performance and gonadal development of the juvenile oriental river prawn (*Macrobrachium nipponense*). Aquacult. Rep..

[20] Zhang Z.P., Shen B.L., Wang Y.L., Chen Y., Wang G.D., Lin P., Zou Z.H. (2010). Molecular cloning of proliferating cell nuclear antigen and its differential expression analysis in the developing ovary and testis of penaeid shrimp *Marsupenaeus japonicus*. DNA Cell Biol..

[21] Okutsu T., Kang B.J., Miwa M., Yoshizaki G., Maeno Y., Wilder M.N. (2010). Molecular cloning and characterization of Dmc1, a gene involved in gametogenesis, from the whiteleg shrimp *Litopenaeus vannamei*. Fish Sci..

[22] Inada M., Mekata T., Sudhakaran R., Okugawa S., Kono T., Asely A.M.E., Linh N.T., Yoshida T., Sakai M., Yui T. (2010). Molecular cloning and characterization of the nitric oxide synthase gene from kuruma shrimp, *Marsupenaeus japonicus*. Fish Shellfish Immunol..

[23] Girish B.P., Swetha C.H., Reddy P.S. (2015). Expression of RXR, EcR, E75 and VtG mRNA levels in the hepatopancreas and ovary of the freshwater edible crab, *Oziothelphusa senex senex* (Fabricius, 1798) during different vitellogenic stages. Naturwissenschaften.

[24] Gong J., Yu K., Shu L., Ye H.H., Li S.J., Zeng C.S. (2015). Evaluating the effects of temperature, salinity, starvation and autotomy on molting success, molting interval and expression of ecdysone receptor in early juvenile mud crabs, *Scylla paramamosain*. J. Exp. Mar. Biol. Ecol..

[25] Li Z., Zhou M.Y., Ruan Y., Chen X.L., Ren C.H., Yang H., Zhang X., Liu J.S., Li H., Zhang L. (2022). Transcriptomic analysis reveals yolk accumulation mechanism from the hepatopancreas to ovary in the Pacific white shrimp *Litopenaeus vannamei*. Front. Mar. Sci..

[26] Tiu S., Benzie H.J., Chan S.M. (2008). From hepatopancreas to ovary: Molecular characterization of a shrimp vitellogenin receptor involved in the processing of vitellogenin. Biol. Reprod..

[27] Gai Z.Q., Wang C., Yang L., Wang W., Deng W., Wu G. (2016). Structural mechanism for the arginine sensing and regulation of CASTOR1 in the mTORC1 signaling pathway. Cell Discov..

[28] Villegas S.N., Gombos R., García-López L., Gutiérrez-Pérez I., García-Castillo J., Vallejo D.M., Da Ros V.G., Ballesta-Illán E., Mihály J., Dominguez M. (2018). PI3K/Akt cooperates with oncogenic notch by inducing nitric oxide-dependent inflammation. Cell Rep..

[29] Budani M.C., Tiboni G.M. (2021). Novel insights on the role of nitric oxide in the ovary: A review of the literature. Int. J. Environ. Res. Public Health.

[30] Li J., Zhou W., Wang Y., Niu C. (2018). The dual role of cGMP in oocyte maturation of zebrafish. Biochem. Biophys. Res. Commun..

[31] Liang X., Luo X., Lin H., Han F., Qin J.G., Chen L., Xu C., Li E. (2022). Effects and mechanism of different phospholipid diets on ovary development in female broodstock Pacific white shrimp, *Litopenaeus vannamei*. Front. Nutr..

[32] Basini G., Grasselli F. (2015). Nitric oxide in follicle development and oocyte competence. Reproduction.

[33] Alborzi P., Jafari Atrabi M., Akbarinejad V., Khanbabaei R., Fathi R. (2020). Incorporation of arginine, glutamine or leucine in culture medium accelerates in vitro activation of primordial follicles in 1-day-old mouse ovary. Zygote.

[34] Grazul-Bilska A.T., Bass C.S., Kaminski S.L., Ebel K.K., Leke E., Thammasiri J., Kraisoon A., Navanukraw C., Holst M., Shelton M. (2019). Effects of plane of nutrition and arginine on ovarian follicles in non-pregnant sheep: Cell proliferation, and expression of endothelial nitric oxide and its receptor. Acta. Histochem..

[35] Dubeibe D.F., Caldas-Bussiere M.C., Maciel V.L., Sampaio W.V., Quirino C.R., Gonçalves P.B., De Cesaro M.P., Faes M.R., Paes de Carvalho C.S. (2017). L-arginine affects the IVM of cattle cumulus-oocyte complexes. Theriogenology.

