# Supplementary material for: Arginine Promoted Ovarian Development in Pacific White Shrimp Litopenaeus vannamei via the NO-sGC-cGMP and TORC1 Signaling Pathways
Source: Animals (Basel). 2024 Jul 5;14(13):1986. doi: 10.3390/ani14131986 (PMC11240395; doi:10.3390/ani14131986)

Table S1. Effects of dietary arginine supplementation ovary amino acids composition in *Litopenaeus vannamei*.

Amino acids	Dietary arginine levels (%)					
	2.90	3.58	4.08	4.53	5.04	5.55
EAA						
Thr	2.53 ± 0.01^{a}	2.5 ± 0.00^{a}	2.58 ± 0.05^{ab}	2.79 ± 0.00^{c}	2.81 ± 0.01^{c}	2.66 ± 0.01^{b}
Val	2.88 ± 0.01^{ab}	3.03 ± 0.11^{abc}	2.81 ± 0.07^{a}	3.14 ± 0.01^{bc}	3.16 ± 0.00^{c}	2.93 ± 0.00^{abc}
Met	0.69 ± 0.05^{b}	0.94 ± 0.01^{c}	0.19 ± 0.10^{a}	0.71 ± 0.02^{b}	0.54 ± 0.07^{b}	0.21 ± 0.00^{a}
Ile	2.24 ± 0.01^{ab}	2.20 ± 0.04^{a}	2.23 ± 0.10^{a}	2.47 ± 0.02^{c}	2.44 ± 0.00^{bc}	2.2 ± 0.00^{a}
Leu	4.01 ± 0.01^{b}	3.76 ± 0.02^{a}	4.25 ± 0.01^{d}	4.42 ± 0.03^{e}	4.11 ± 0.00^{c}	3.71 ± 0.01^{a}
Phe	2.52 ± 0.03^{b}	2.13 ± 0.01^{a}	2.47 ± 0.08^{b}	2.74 ± 0.03^{c}	2.57 ± 0.00^{bc}	2.39 ± 0.01^{b}
Lys	4.04 ± 0.00^{c}	3.30 ± 0.01^{a}	4.24 ± 0.01^{d}	4.44 ± 0.02^{e}	4.08 ± 0.00^{c}	3.62 ± 0.01^{b}
His	1.43 ± 0.03^{c}	1.23 ± 0.01^{a}	1.46 ± 0^{cd}	1.53 ± 0.01^{e}	1.49 ± 0.00^{de}	1.35 ± 0.01^{b}
Arg	4.10 ± 0.01^{d}	2.87 ± 0.03^{a}	4.47 ± 0.01^{e}	4.52 ± 0.03^{e}	3.81 ± 0.00^{c}	3.31 ± 0.00^{b}
NEAA						
Asp	5.37 ± 0.03^{a}	5.42 ± 0.09^{ab}	5.26 ± 0.22^{a}	5.85 ± 0.03^{b}	5.39 ± 0.01^{ab}	4.98 ± 0.00^{a}
Ser	2.42 ± 0.01^{ab}	2.33 ± 0.01^{a}	2.41 ± 0.06^{ab}	2.61 ± 0.01^{c}	2.52 ± 0.01^{bc}	2.32 ± 0.00^{a}
Glu	8.17 ± 0.01^{b}	8.79 ± 0.02^{d}	8.87 ± 0.01^{d}	9.00 ± 0.05^{e}	8.30 ± 0.01^{c}	7.21 ± 0.01^{a}
Gly	3.64 ± 0.01^{e}	3.07 ± 0.01^{c}	2.97 ± 0.02^{b}	3.93 ± 0.01^{f}	3.24 ± 0.01^{d}	2.73 ± 0.00^{a}
Ala	3.01 ± 0.02^{b}	3.25 ± 0.01^{cd}	3.21 ± 0.01^{c}	3.31 ± 0.01^{e}	3.27 ± 0.00^{d}	2.90 ± 0.01^{a}
Cys	0.51 ± 0.01^{abc}	0.49 ± 0.02^{ab}	0.48 ± 0.01^{a}	0.56 ± 0.01^{c}	0.55 ± 0.01^{bc}	0.49 ± 0.01^{ab}
Tyr	1.91 ± 0.04	1.93 ± 0.05	1.88 ± 0.05	2.10 ± 0.05	1.88 ± 0.01	1.69 ± 0.00
Pro	2.70 ± 0.05^{b}	2.35 ± 0.08^{a}	2.72 ± 0.10^{b}	2.90 ± 0.02^{b}	2.81 ± 0.02^{b}	2.34 ± 0.00^{a}
∑EAA	24.43 ± 0.12^{b}	21.96 ± 0.16^{a}	24.72 ± 0.41^{b}	26.75 ± 0.17^{c}	25.00 ± 0.08^{b}	22.38 ± 0.05^{a}
∑NEAA	27.73 ± 0.15^{a}	27.63 ± 0.05^{a}	27.81 ± 0.47^{a}	30.25 ± 0.14^{c}	27.96 ± 0.10^{a}	24.66 ± 0.03^{a}
TAA	52.16 ± 0.27^{c}	49.59 ± 0.21^{b}	52.53 ± 0.88^{c}	56.99 ± 0.3^{d}	52.96 ± 0.17^{c}	47.04 ± 0.08^{a}
∑EAA/TAA	0.47 ± 0.00^{b}	0.44 ± 0.00^{a}	0.47 ± 0.00^{bc}	0.47 ± 0.00^{b}	0.47 ± 0.00^{c}	0.48 ± 0.00^{d}

Table S2. Effects of dietary arginine supplementation hepatopancreas amino acids composition in *Litopenaeus vannamei*.

Amino acids	Dietary arginine levels (%)					
	2.90	3.58	4.08	4.53	5.04	5.55
EAA						
Thr	1.66 ± 0.01^{b}	1.71 ± 0.02^{bc}	1.45 ± 0.05^{a}	1.78 ± 0.01^{c}	1.42 ± 0.01^{a}	1.67 ± 0.01^{bc}
Val	1.86 ± 0.02^{b}	1.94 ± 0.03^{bc}	1.64 ± 0.06^{a}	2.02 ± 0.02^{c}	1.67 ± 0.02^{a}	1.85 ± 0.00^{b}
Met	0.58 ± 0.01	0.50 ± 0.01	0.42 ± 0.04	0.53 ± 0.12	0.48 ± 0.02	0.45 ± 0.03
Ile	1.38 ± 0.01^{b}	1.43 ± 0.02^{b}	1.21 ± 0.05^{a}	1.50 ± 0.03^{b}	1.23 ± 0.02^{a}	1.38 ± 0.00^{b}
Leu	2.39 ± 0.02^{b}	2.47 ± 0.05^{bc}	2.12 ± 0.06^{a}	2.61 ± 0.06^{c}	2.13 ± 0.04^{a}	2.45 ± 0.02^{bc}
Phe	1.38 ± 0.02^{ab}	1.44 ± 0.03^{ab}	1.23 ± 0.03^{a}	1.56 ± 0.06^{ab}	1.50 ± 0.11^{b}	1.41 ± 0.00^{b}
Lys	2.06 ± 0.01^{bc}	2.15 ± 0.03^{cd}	1.85 ± 0.02^{a}	2.29 ± 0.07^{d}	1.97 ± 0.03^{ab}	2.21 ± 0.01
His	0.77 ± 0.01^{b}	0.8 ± 0.01^{bc}	0.68 ± 0.01^{a}	0.81 ± 0.00^{c}	0.69 ± 0.01^{a}	0.79 ± 0.00^{bc}
Arg	1.85 ± 0.01^{ab}	1.93 ± 0.05^{bc}	1.76 ± 0.01^{a}	2.06 ± 0.03^{c}	1.78 ± 0.01^{a}	2.02 ± 0.01^{c}
NEAA						
Asp	3.51 ± 0.04^{bc}	3.64 ± 0.03^{bc}	3.01 ± 0.14^{a}	3.75 ± 0.01^{c}	2.92 ± 0.02^{a}	3.46 ± 0.01^{b}
Ser	1.56 ± 0.01^{b}	1.60 ± 0.03^{bc}	1.35 ± 0.04^{a}	1.68 ± 0.00^{c}	1.32 ± 0.02^{a}	1.55 ± 0.01^{b}
Glu	4.27 ± 0.00^{bc}	4.45 ± 0.08^{c}	4.09 ± 0.04^{b}	4.73 ± 0.00^{d}	3.77 ± 0.04^{a}	4.4 ± 0.03^{c}
Gly	2.05 ± 0.04^{bc}	2.08 ± 0.04^{cd}	1.90 ± 0.06^{ab}	2.25 ± 0.03^{d}	1.79 ± 0.01^{a}	2.01 ± 0.01^{bc}
Ala	1.85 ± 0.01^{a}	1.91 ± 0.04^{a}	1.85 ± 0.03^{a}	2.03 ± 0.01^{c}	1.68 ± 0.03^{a}	1.87 ± 0.01^{a}
Cys	0.32 ± 0.01	0.28 ± 0.02	0.22 ± 0.00	0.27 ± 0.06	0.26 ± 0.01	0.21 ± 0.01
Tyr	1.23 ± 0.03^{ab}	1.24 ± 0.01^{ab}	1.08 ± 0.02^{a}	1.36 ± 0.07^{b}	1.24 ± 0.07^{ab}	1.19 ± 0.00^{ab}
Pro	1.76 ± 0.01^{ab}	1.73 ± 0.07^{ab}	1.71 ± 0.00^{a}	1.86 ± 0.01^{b}	1.69 ± 0.01^{a}	1.79 ± 0.00^{ab}
∑EAA	13.93 ± 0.11^{bc}	14.36 ± 0.25^{cd}	12.37 ± 0.23^{a}	15.16 ± 0.40^{d}	12.87 ± 0.25^{ab}	14.22 ± 0.03^{cd}
∑NEAA	16.55 ± 0.13^{b}	16.90 ± 0.27^{b}	15.2 ± 0.33^{a}	17.92 ± 0.11^{c}	14.68 ± 0.02^{a}	16.47 ± 0.04^{b}
TAA	30.48 ± 0.24^{b}	31.27 ± 0.52^{b}	27.57 ± 0.56^{a}	33.08 ± 0.52^{c}	27.54 ± 0.27^{a}	30.69 ± 0.07^{b}
∑EAA/TAA	0.46 ± 0.00^{ab}	0.46 ± 0.00^{ab}	0.45 ± 0.00^{a}	0.46 ± 0.01^{ab}	0.47 ± 0.00^{b}	0.46 ± 0.00^{b}

Figure S1: The result of dsGFP, dsTORC1 and dsNOS

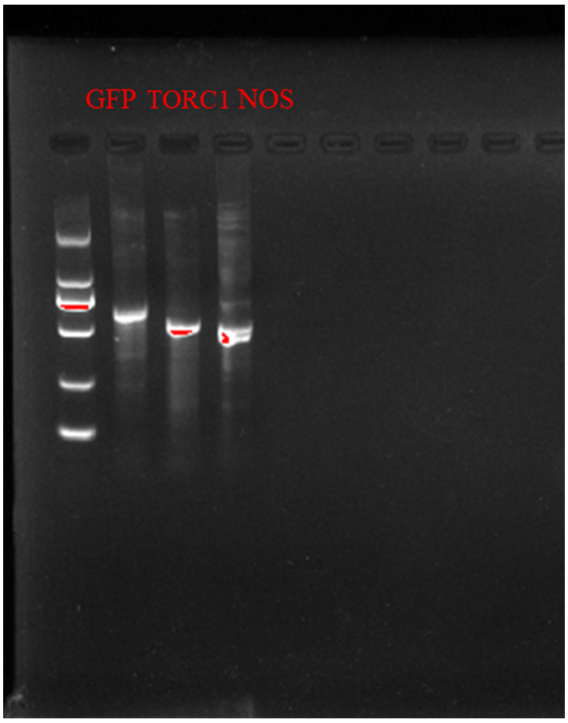

Supplement: Supplementary file 1 [file animals-14-01986-s001.zip › animals-2985473-supplementary.pdf]
